# Robust MPC for polytopic uncertain systems *via* a high-rate network with the round-robin scheduling

**DOI:** 10.7717/peerj-cs.1269

**Published:** 2023-03-10

**Authors:** Jianhua Wang, Yiling Wang, Xialai Wu, Wenyan Ci

**Affiliations:** School of Engineering, Huzhou University, Huzhou, Zhejiang, China

**Keywords:** RMPC, High-rate communication, Polytopic uncertain systems, RR scheduling, Token-dependent Lyapunov-like approach

## Abstract

This article is concerned with the robust model predictive control (RMPC) problem for polytopic uncertain systems under the round-robin (RR) scheduling in the high-rate communication channel. From a set of sensors to the controller, several sensors transmit the data to the remote controller *via* a shared high-rate communication network, data collision might happen if these sensors start transmissions at the same time. For the sake of preventing data collision in the high-rate communication channel, a communication scheduling known as RR is used to arrange the data transmission order, where only one node with token is allowed to send data at each transmission instant. In accordance with the token-dependent Lyapunov-like approach, the aim of the problem addressed is to design a set of controllers in the framework of RMPC such that the asymptotical stability of the closed-loop system is guaranteed. By taking the effect of the underlying RR scheduling in the high-rate communication channel into consideration, sufficient conditions are obtained by solving a terminal constraint set of an auxiliary optimization problem. In addition, an algorithm including both off-line and online parts is provided to find a sub-optimal solution. Finally, two simulation examples are used to demonstrate the usefulness and effectiveness of the proposed RMPC strategy.

## Introduction and Literature Review

Over the past several decades, model predictive control (MPC), also called receding horizon control (RHC) has attracted extensive research and applications, see (Li et al., 2015; Dai et al., 2018; Domina & Tihanyi, 2022). At each time instant, multiple control moves are calculated by solving a fixed number of online optimization problems in the future time instants, but only the first action is executed. At the next sampling time instant, the control input needs to be calculated by reconstructing the optimization problem with the new measurements obtained from the system. Also, according to the mechanism characteristics of MPC, mounts of excellent research results of MPC method exist in a large number of literatures, see *e.g*., (Li et al., 2016; Xia et al., 2010; Zou et al., 2015; Li, Yan & Shi, 2018; Song et al., 2022). However, such as the MPC strategy in Li et al. (2015) may not be effective for nominal systems with parameter uncertainties. Therefore, some extensions are made to the inevitable parameter uncertainties (Song et al., 2016; Zhu, Song & Ding, 2018; Wang & Song, 2016) in the modeling process, which brings about the so-called robust MPC (RMPC) method (see *e.g*., Kothare, Balakrishnan & Morari, 1996; Zhang et al., 2008; Wang, Song & Wei, 2021; Wang, Song & Wei, 2020; Liu et al., 2018).

Generally speaking, many works assume that the system state can be measured online. However, in practical application, the states of the system cannot be always obtained in actual time, so the RMPC strategies relying on the system state may be to no avail. The synthesis methods of output feedback RMPC are thus generated. In Wan & Kothare (2002), an off-line output-feedback RMPC is given. In Shi, Lu & Qiang (2016), the problem of output-feedback RMPC for a class of linear uncertain systems is concerned with, which ensure the stability of the uncertain systems. In Wang et al. (2016), the network security problem has been studied on the basis of the static output-feedback-based RMPC. Hence, this constitutes our primary purpose to propose a static output-feedback RMPC strategy by dealing with the side effects of the immeasurable state.

Along with the development of the communication technology and the computer science, network connection has been used in the actual systems widely, because it has advantages like power requirements and easy maintenance, see *e.g*., (Wang et al., 2022; Zou et al., 2021; Zhu et al., 2021; Cao & Zhu, 2022). The control system with components connected *via* a network is called a networked control system (NCS). In the NCS, a high-rate communication network is characterized by that the transmission rate of signals over the network channel is sometimes much faster than the sampling rate of sensors. For example, in Zou et al. (2017b), communication networks such as process field bus are called high-rate communication networks, which will lead to the special situation that sensors transmit the same measurement output multiple times in one sampling period.

Although the data transmission speed of high-rate communication channels is very fast, it is likely to lead to data conflict due to the simultaneous transmissions of multiple sensors. Therefore, in Shen et al. (2020), in order to alleviate the data conflict, a communication scheduling is needed, which can schedule the sensor transmission sequence according to certain principles adopted in the high-rate communication channel. That is to say, to prevent data conflict, some communication schedulings are more and more used in the networked system, including round-robin (RR) scheduling in Zou, Wang & Gao (2016b), Wang, Song & Wei (2019), Bauer et al. (2013), Donkers et al. (2011), Liu, Fridman & Hetel (2012), Zhu et al. (2018), try-once-discard scheduling in Zou, Wang & Gao (2016b), Song et al. (2019), stochastic communication scheduling in Zou, Wang & Gao (2016a), Zou et al. (2017a), Song et al. (2020). In Ding et al. (2019), among numerous communication schedulings, round robin (RR) scheduling is a periodic communication scheduling, which regulates the transmission access of sensors according to a fixed cyclic sequence, and is extensively used in industry. Compared with dynamic protocols, static scheduling protocols like RR protocol can handle the data collision with less resource-consuming and easy-to-implementation, so it is adopted during the data communication of the addressed NCSs. However, such as the discussion (Liu, Fridman & Hetel, 2012), it is difficult to cope with the data orchestration for the polytopic uncertain systems. Therefore, it should be noted that the introduction of RR scheduling in high-rate communication channels will further complicate the signal transmission behavior and bring substantive difficulties to the design of corresponding controller. In addition, such as the description in Zhu et al. (2018), the performance might be degraded since only a part of the information can be successfully updated at every time instant due to the RR protocol. It is therefore significantly important to take the influence of the RR protocol adequately into the consideration so as to reduce its side-effects, which is our primary motivation of this article. To this day, despite the important engineering significance, the RMPC problem for the polytopic uncertain systems under high-rate communication channels has not been investigated extensively yet. Therefore, the RMPC problem based on RR scheduling in high-rate communication channels is an interesting and challenging research problem.

We need to dispose of some challenging issues:

1. how to consider the influences of the RR scheduling in the high-rate communication channels fully to reduce the side effects of the underlying scheduling,
2. how to face or deal with the problem that complicates the signal transmission behavior,
3. how to ensure the asymptotical stability of the closed-loop system and the recursive feasibility of the algorithm,
4. how to guarantee the feasibility of the matrix inequality, which is generated by synthesizing the RR scheduling and the high-rate communication channels.

In this article, some contributions are as follows:

1. a comprehensive model is put forward which takes the high-rate communication channels, the RR scheduling and polytopic uncertainties into simultaneous consideration, thereby better reflecting the reality;
2. the data transmission problem is studied for the RMPC under high-rate communication channels with RR scheduling;
3. based on the RMPC strategy, the controller design reflects the effects of the polytopic uncertainties, the underlying RR scheduling and the high-rate communication channels;
4. due to the high-rate communication channels with RR scheduling and Lyapunov-like approach, a set of controllers in the framework of RMPC are designed by solving the online optimization problem, with which the system state is stabilized to the origin quickly.

The aim of this study is to design static output-feedback controllers within the framework of RMPC, which can make the system asymptotically stable under the RR scheduling in high-rate communication channels. The RR scheduling is used to prevent data collision. The application of high-rate communication channels is to improve prediction performance. Specifically, based on the token-dependent Lyapunov-like approach, some auxiliary optimization problems are provided to find the required parameter matrices. The results showed that the stability of the closed-loop system is guaranteed well.

## Problem Statement and Preliminaries

In this part, for systems with polytopic uncertainties under the RR scheduling in the high-rate communication channel, the switching system is produced. For the underlying system, a static output-feedback RMPC problem is established. In addition, many preparations are made for the following performance analysis. In this article, the notations are standard as shown in Table 1.

**Table 1:** A symbol table.

Symbol	Denotation
${\mathbb R}^{n}$	The $n$-dimensional Euclidean space
${\mathbb R}^{n \times m}$	The set of all $n \times m$ real matrices
$P \gt 0$	The symmetric and positive definite matrix
$||x||$	The Euclidean norm and $||x||^{2} = {x^T}x$
$|| \cdot ||{_F}$	The Frobenius norm of ${\mathbb R}^{n}$ space
$|a|$	The absolute value of a
*I*	The identity matrix with appropriate dimensions
$0$	The zero matrix with appropriate dimensions
${M^T}$	The transpose of a real matrix *M*
${M^{ - 1}}$	The inverse (if invertible) of a real matrix *M*
*	The symmetric part of the symmetric matrix
diag $\{ \cdot \cdot \cdot \}$	The block-diagonal matrix

### System models

Let us consider the discrete-time linear polytopic uncertain systems:

(1) $$\left\{ {\matrix{ {x({t_{k + 1}}) = A({t_k})x({t_k}) + B({t_k})u({t_k})} \hfill \cr  {y({t_k}) = C({t_k})x({t_k})} \hfill \cr  } } \right.$$

where ${t_k}(k\geq0)$ represents the $(k + 1)$th sampling instant, $x({t_k}) \in {\mathbb R}^{{n_x}}$, $u({t_k}) \in {\mathbb R}^{{n_u}}$ and $y({t_k}) \in {\mathbb R}^{{n_y}}$ are the state, the control input and the measurement output, respectively. $A({t_k})$, $B({t_k})$ and $C({t_k})$ with appropriate dimensions are unknown matrices, which belong to a polytope given by

(2) $$\Delta : = \{ \Theta |\Theta = \sum\limits_{l = 1}^L {{\lambda _l}} {\Theta ^{(l)}},\sum\limits_{l = 1}^L {{\lambda _l}} = 1,0 \le {\lambda _l} \le 1\} ,$$

where $\Theta : = (A({t_k}),B({t_k}),C({t_k})) \in \Delta$, and matrices ${\Theta ^{(l)}}$ are previously known and defined as ${\Theta ^{(l)}}: = ({A^{(l)}},{B^{(l)}},{C^{(l)}}),l = 1,2, \ldots ,L$, which are the vertices in the convex hull $\Delta$.

In engineering systems, consider the actual applicability of the strategy that we propose, the constraints on the inputs and states are given by

(3) $$\left\{ {\matrix{ {\mathop {\max }\limits_c |{{[u({t_k})]}_c}| \le \bar u,\quad c \in \{ 1,2,\ldots,{n_u}\} } \cr {\mathop {\max }\limits_d |{{[x({t_k})]}_d}| \le \bar x,\quad d \in \{ 1,2,\ldots,{n_x}\} } } } \right.$$

where ${[.]_j}$ denotes the $j$th element of a vector, $j \in \{ c,d\}$. $\bar u \gt 0$, $\bar x \gt 0$ are known scalars.

### Communication schedulings

**Figure 1:**
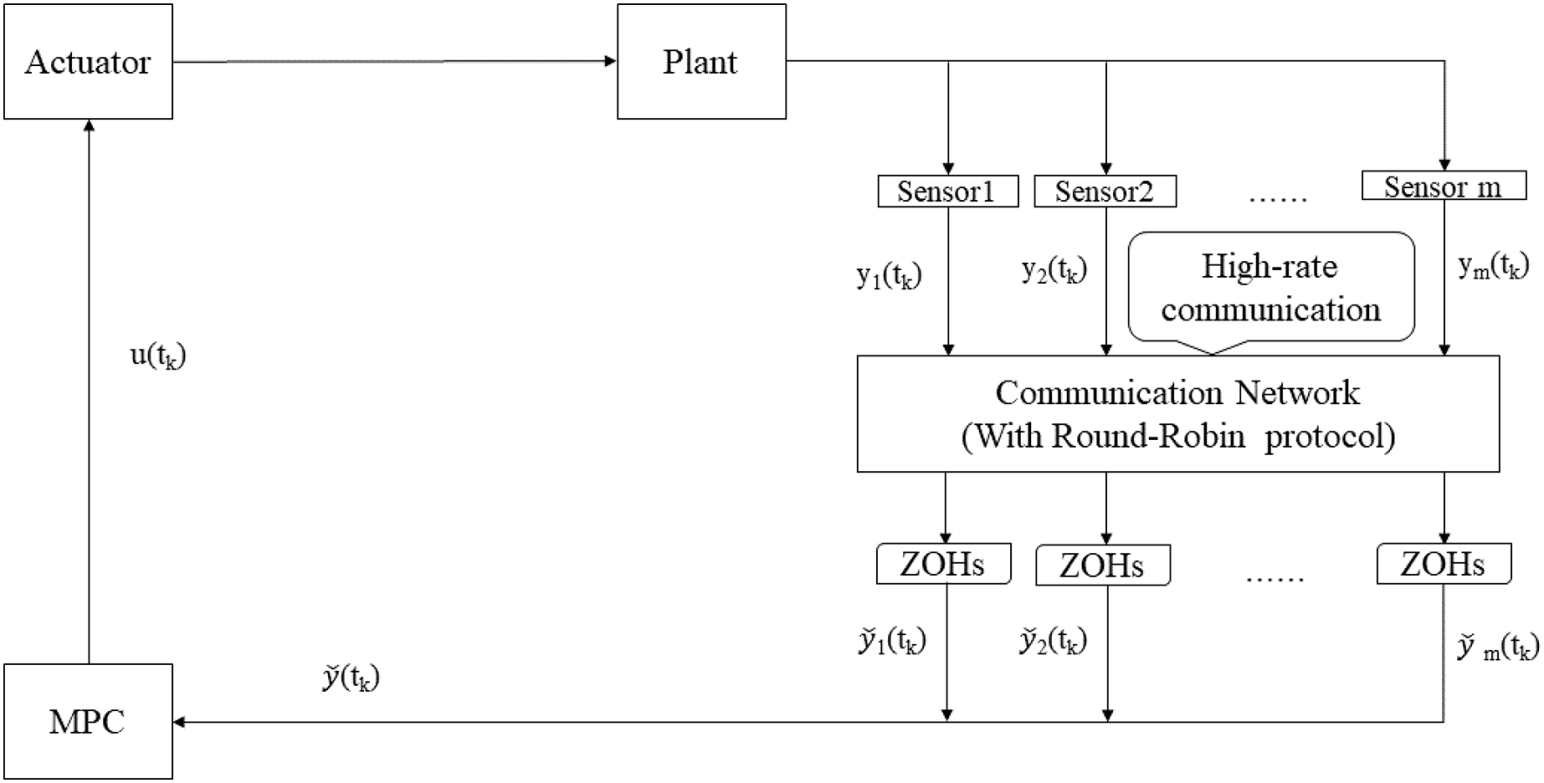
Structure of RMPC-based system under RR scheduling.

In Fig. 1, the data are transmitted by the sensors to the controller *via* a shared network with high-rate communication channels. For the sake of reducing the network transmission burden and avoiding congestion, a famous RR scheduling is used to arrange the data transmission sequence. To be more specific, only one sensor node with the permission has the access to the shared network, while others maintain the previous information by zero-order-holders (ZOHs). In particular, under the RR scheduling, data are transmitted periodically in cyclic order according to a predetermined scheduling principle.

Let ${\hat y_i}({t_k})$ mean the measurement obtained from sensor $i$. The update of ${\hat y_i}({t_k})$ under the RR scheduling can be denoted by

(4) $${\hat y_i}({t_k}) = \left\{ {\matrix{ {{y_i}({t_k}),} \hfill & {if\;mod({t_{k - i}},m) = 0} \hfill \cr  {{{\hat y}_i}({t_{k - 1}}),} \hfill & {otherwise} \hfill \cr  } } \right.$$

The combination of ${\hat y_i}({t_k})$ $(i = 1,2,3,...,m)$ means the information obtained from the controller at the time instant ${t_k}$, ${\hat y_i}({t_k})$ is defined by $\hat y({t_k}) \buildrel \Delta \over = {[\hat y_1^T({t_k}),\hat y_2^T({t_k}),\ldots,\hat y_m^T({t_k})]^T}$.

$Remark\;1.$ In this article, the measurement outputs of the sensors are transmitted to the remote controller *via* the high-rate communication channels. Because a large number of sensors are deployed, the data collision occur when sensors transmit the measurement signals at the same time. In order to prevent data conflict, the RR scheduling is used to determine the transmission sequence of sensors in high-rate communication channels. Before the further discussion, the following assumption on the high-rate communication channels is made.

$Assumption\;1.$ The time interval between two adjacent transmissions in the high-rate communication channels is ${t_h} = {t_s}/d$ with $d\geq1$ being a positive integer. The sampling period of the sensors is denoted as ${t_s} \buildrel \Delta \over = {t_{k + 1}} - {t_k}$. In addition, the first transmission occurs at the initial sampling instant ${t_0}$.

Denoting the $(k^{\prime} + 1)$th $(k^{\prime}\geq0)$ transmission time instant as $T_k^{\prime}$, we have $T_{k^{\prime} + 1} = T_{k^{\prime} }+ {t_h}$. In addition, it is easy to know the measurement output sent by sensor $i$ at time instant $T_{k^{\prime}}$ is

(5) $${y_i}(T_{k^{\prime}}) = {y_i}({t_k}),\quad T_{k^{\prime}} \in [{t_k},{t_{k + 1}}).$$

Now, we are going to explain the scheduling effect of RR scheduling in high-rate communication channels in detail. Let $\check{y_i}(T_{k^{\prime}})$ be the actual received measurement output from sensor $i$ at time instant $T_{k^{\prime}}$. From the knowledge of the RR scheduling, we have

(6) $${\check y_i}({T_{k^{\prime}}}) = \left\{ \matrix{ {y_i}({T_{k^{\prime}}}),\quad if\;\xi ({T_{k^{\prime}}}) = i;{\rm{ }} \hfill \cr {\check y_i}({T_{k^{\prime} - 1}}),\quad otherwise \hfill \cr} \right.$$

where $\xi (T_{k^{\prime}}) \buildrel \Delta \over = mod(k^{\prime} - 1,n) + 1$ represents the sensor which is given the transmission access at time instant $T_{k^{\prime}}$. Moreover, we set $\check{y_i}(T_{k^{\prime}}) = 0$ for any $k^{\prime}{\rm{\lt}}\;0$. Denote

$$\check y(T_{k^{\prime}}) \buildrel \Delta \over = col\{ \check{y_1}(T_{k^{\prime}}),\check{y_2}(T_{k^{\prime}}),\ldots,\check{y_n}(T_{k^{\prime}})\} ,$$

$$y({t_k}) \buildrel \Delta \over = col\{ {y_1}({t_k}),{y_2}({t_k}),\ldots,{y_n}({t_k})\} ,$$

$$\Psi _{\xi (T_{k^{\prime}})} \buildrel \Delta \over = diag\{ \delta (\xi (T_{k^{\prime}}),1)I,\delta (\xi (T_{k^{\prime}}),2)I,\ldots,\delta (\xi (T_{k^{\prime}}),n)I\}$$

with $\delta$ (.,.) being the Kronecker delta function. From Eqs. (5) and (6), one has

(7) $$\check y(T_{k^{\prime}}) = \Psi_{\xi (T_{k^{\prime}})}y({t_k}) + (I - \Psi_{\xi (T_{k^{\prime}}))}\check y(T_{k^{\prime} -1}),\quad T_{k^{\prime}} \in [{t_k},{t_{k + 1}}).$$

From Assumption 1, we know that there are $d$ transmissions occurring in the time interval $({t_{k - 1}},{t_k}]$. Denote the time instant when the $\beta$th transmission in the time interval $({t_{k - 1}},{t_k}]$ occurs as $\bar t_k^\beta \buildrel \Delta \over = {t_{k - 1}} + \beta {t_h}(\beta = 1,2,...,d)$. From Eq. (7), we have

$$\check y({t_k}) = {\Psi _{\xi ({t_k})}}y({t_k}) + (I - {\Psi _{\xi ({t_k})}})\check y(\bar t_k^{d - 1}),$$

$$\check y(\bar t_k^{d - 1}) = {\Psi _{\xi (\bar t_k^{d - 1})}}y({t_{k - 1}}) + (I - {\Psi _{\xi (\bar t_k^{d - 1})}})\check y(\bar t_k^{d - 2}),$$

$$\vdots$$

$$\check y(\bar t_k^1) = {\Psi _{\xi (\bar t_k^1)}}y({t_{k - 1}}) + (I - {\Psi _{\xi (\bar t_k^1)}})\check y({t_{k - 1}}).$$

By iterating the above equations, we further obtain

(8) $$\check y({t_k}) = {\Psi _{\xi ({t_k})}}y({t_k}) + \phi _{\zeta (\bar t_k^d)}^1y({t_{k - 1}}) + \phi _{\zeta (\bar t_k^d)}^2\check y({t_{k - 1}})$$

where

$$\zeta (\bar t_k^d) \buildrel \Delta \over = (\xi (\bar t_k^1),\xi (\bar t_k^2),\ldots,\xi (\bar t_k^d)),$$

$$\phi _{\zeta (\bar t_k^d)}^1 \buildrel \Delta \over = \Sigma _{i = 1}^{d - 1}(\Pi _{j = 0}^{i - 1}(I - {\Psi _{\xi (\bar t_k^{d - j})}})){\Psi _{\xi (\bar t_k^{d - i})}},$$

$$\phi _{\zeta (\bar t_k^d)}^2 \buildrel \Delta \over = \Pi _{i = 0}^{d - 1}(I - {\Psi _{\xi (\bar t_k^{d - i})}}).$$

$Remark\;2.$ It can be seen that the matrix ${X_{\zeta (\bar t_k^d)}}(X$ stands for ${\phi ^1},{\phi ^2},{\phi ^3},\bar \Psi$ and ${\bar \Psi ^i})$ is dependent on $\xi (\bar t_k^1),\xi (\bar t_k^2),\ldots,\xi (\bar t_k^d)$ and therefore ${X_{\zeta (\bar t_k^d)}}$ should be written as ${X_{\xi (\bar t_k^1),\xi (\bar t_k^2),\ldots,\xi (\bar t_k^d)}}$. However, for convenience of presentation, we introduce a new notation $\zeta (\bar t_k^d)$ to represent $\xi (\bar t_k^1),\xi (\bar t_k^2),\ldots,\xi (\bar t_k^d)$, and ${X_{\xi (\bar t_k^1),\xi (\bar t_k^2),\ldots,\xi (\bar t_k^d)}}$ is represented by ${X_{\zeta (\bar t_k^d)}}$.

### Problem of interests

Because it is difficult to get the states of the system in actual practice, for the underlying system (Eq. (1)), the following static output-feedback controller based on MPC is designed under the RR scheduling in the high-rate communication channel:

(9) $$u({t_k}) = {F_{\xi ({t_k})}}({t_k})\check y({t_k}),$$

${F_{\xi ({t_k})}}({t_k})$ is the feedback gain to be designed by optimization.

On the basis of the control law (Eq. (9)), we can adapt the closed-loop system which is on the prediction horizon as the following:

(10) $$\chi ({t_{k + 1}}) = {\bar A_{\xi ({t_{k + n}}|k)}}({t_k})\chi ({t_k}),$$

where

$$\chi ({t_k}) \buildrel \Delta \over = col\{ x({t_k}),y({t_{k - 1}}),\check y({t_{k - 1}})\} ,$$

$${\bar A_{\xi ({t_{k + n}}|k)}}({t_k}) = \left[ {\matrix{ {A({t_k}) + B({t_k}){F_{\xi ({t_k})}}{\Psi _{\xi ({t_k})}}C({t_k})}  &  {B({t_k}){F_{\xi ({t_k})}}\Phi _{\zeta (\bar t_k^d)}^1}  &  {B({t_k}){F_{\xi ({t_k})}}\Phi _{\zeta (\bar t_k^d)}^2} \cr {C({t_k})}  &  0  &  {0} \cr {{\Psi _{\xi ({t_k})}}C({t_k})}  &  {\Phi _{\zeta (\bar t_k^d)}^1}  &  {\Phi _{\zeta (\bar t_k^d)}^2} \cr } } \right]$$

The initial state is obtained by $\chi (0) = col\{ x(0)\;y( - 1)\;\check y( - 1)\}$.

For the system (Eq. (10)) with polyhedral parameter uncertainties, the “min-max” problem over an infinite time horizon is used to design the controllers:

(11) $$\mathop {\min }\limits_{{F_{\xi ({t_{k + n}}|k)}}} \mathop {\max }\limits_{(A({t_k}),B({t_k}),C({t_k})) \in \Delta } {J_\infty }({t_k}),$$

we define the objective function ${J_\infty }({t_k})$ by

(12) $${J_\infty }({t_k}) \buildrel \Delta \over = \mathop \sum \limits_{n = 0}^\infty \left[ {{\chi ^T}({t_k})Q\chi ({t_k}) + {u^T}({t_k})Ru({t_k})} \right].$$

${Q_1},{Q_2},{Q_3}$ and *R* denote the symmetric and positive definite weighting matrices, and $Q = diag\{ {Q_1},{Q_2},{Q_3}\}$.

Based on the min-max problem (Eq. (12)), for the aim of designing the controllers in the framework of out-feedback MPC, we present the online optimization problem:

$${\rm{Op1}}:\mathop {\min }\limits_{{F_{\xi ({t_{k + n}}|k)}}} \mathop {\max }\limits_{(A({t_k}),B({t_k}),C({t_k})) \in \Delta } {J_\infty }({t_k}),$$

$${\rm{s}}{\rm{.t}}{\rm{.}}\quad {\max _c}|{[u({t_k})]_c}| \le \bar u,$$

$${\rm{s}}{\rm{.t}}{\rm{.}}\quad {\max _d}|{[x({t_k})]_d}| \le \bar x,$$

$$\chi ({t_k}) \in \Upsilon ({P_{\xi ({t_{k + n}}|k)}},3\rho ),$$

where $\Upsilon$ is what is called a terminal constraint set, we define it by

(13) $$\Upsilon \buildrel \Delta \over = \{ \chi({t_k})|\chi {^T}({t_k}){P_{\xi ({t_{k + n}}|k)}}\chi({t_k})\leq3\rho \}$$

${P_{\xi ({t_{k + n}}|k)}}$ denotes a positive definite matrix of aquadratic function. We will present more details in the next section. Note that only the first component $u({t_k})$ of predicted inputs $\{ u({t_k}),u({t_{k + 1}}),u({t_{k + 2}}),...\}$ will be worked on the plant at each time instant.

In this article, the static output-feedback controllers (Eq. (9)) are designed within the framework of RMPC to make the system (Eq. (1)) asymptotically stable under RR scheduling (Eq. (4)) in high-rate communication channels. To be more specific, an auxiliary optimization problem Op1 is provided to find the required parameter matrices ${F_{\xi ({t_k})}}$, so that we can guarantee the stability of the closed-loop system. In order to reach this goal, we need to satisfy the following requirements for any admissible parameter $k$ at the same time:

$R1.$ an auxiliary optimization issue is supplied to denote the problem Op1, so that we can get the suboptimal solution;

$R2.$ according to the parameter matrices ${F_{\xi ({t_k})}}$ that we obtain, the closed-loop system under the RR scheduling (Eq. (4)) in high-rate communication channels is asymptotically stable.

## Main Results

### MPC under RR scheduling without hard constraints

This section will provide several sufficient conditions for the systems without constraints to ensure the desired performance through the quadratic function method. Accordingly, we obtain the static output-feedback controllers under the RMPC framework. To be exact, firstly, sufficient conditions are given to meet the condition of the terminal constraint set in Op1, *i.e*., $\chi ({t_k}) \in \Upsilon ({P_{\xi ({t_{k + n}}|k)}},3\rho )$. After that, an auxiliary optimization problem is proposed to find the suboptimal solution for the unconstrained system. In addition, the inequation analysis technology is used to deal with the unavailable state $x({t_k})$ problem of the auxiliary problem, we provide another auxiliary problem for the solvability. In the end, by solving this kind of online auxiliary optimization issue, we obtain sufficient conditions to guarantee the stability of the closed-loop system.

### Terminal constraint set

Before the main results are developed, we first present the significant definition.

$Definition\;1.$ For system (Eq. (1)) under the control law (Eq. (9)), the set $\Upsilon$ is a robust positive invariant (RPI) set if $\chi ({t_k}) \in \Upsilon$ implies $\chi ({t_{k + 1}}) \in \Upsilon$.

Based on the online optimization problem Op1, we need to satisfy the following two conditions such that the set $\Upsilon ({P_{\xi ({t_{k + n}}|k)}},3\rho )$ is the terminal constraint set for Op1:

C1: we define a quadratic function by

(14) $$V(\chi ({t_{k + n}}|k)) \buildrel \Delta \over = {\chi ^T}({t_{k + n}}|k){P_{\xi ({t_{k + n}}|k)}}\chi ({t_{k + n}}|k),$$

such that

$$V = V(\chi ({t_{k + n + 1}}|k)) - V(\chi ({t_{k + n}}|k))$$

(15) $$V{\leq} - \chi{^T}({t_{k + n}}|k){\rm{\} }}Q\chi({t_{k + n}}|k){\rm{\} }} - {u^T}({t_{k + n}}|k)Ru({t_{k + n}}|k).$$

C2: the set $\Upsilon ({P_{\xi ({t_{k + n}}|k)}},3\rho )$ is an RPI set.

Next, we will discuss the above conditions. For the sake of simplicity, we define $\xi ({t_{k + n}}|k) = r$, $\xi ({t_{k + n + 1}}|k) = t$.

The following theorem is shown to ensure the condition C1 of terminal constraint set.

$Lemma\;1.$ Let us give symmetric positive definite matrices ${Q_1}$, ${Q_2}$, ${Q_3}$ and *R*. For the system (Eq. (10)) which is controlled by Eq. (9), if there is a positive scalar $\rho \gt 0$, symmetric and positive definite matrices ${\tilde Q_{ir}}$, ${\tilde Q_{it}}(i = 1,2,3)$ and matrices ${Y_r}$, for any $(r,t) \in \mathbb S \times \mathbb S$, $l = 1,2,...,L$, such that the following conditions are established:

(16) $$\left[ {\matrix{ {\hat Q_r^l}  &  *  &  *  &  * \cr  {\hat A_r^l}  &  {{{\tilde Q}_t}}  &  *  &  * \cr  {\sqrt R \bar M}  &  0  &  {\rho I}  &  * \cr  \Xi  &  0  &  0  &  {\bar \rho I} \cr  } } \right]\quad \ge 0,$$

where

$$\bar \rho I = \left[ {\matrix{ {\rho I}  &  0  &  {0} \cr 0  &  {\rho I}  &  {0} \cr 0  &  0  &  {\rho I} \cr } } \right],\quad$$

$$\bar M = {F_r}\left[ {\matrix{ {{\Psi _{\xi ({t_k})}}{C^l}{T^l}S}  &  {\Phi _{\zeta (\bar t_k^d)}^1{S_{11}}}  &  {\Phi _{\zeta (\bar t_k^d)}^2{S_{11}}} \cr } } \right],\quad$$

$$\hat Q_r^l = \left[ {\matrix{ {{{\bar Q}_{1r}}}  &  0  &  {0} \cr 0  &  {{{\bar Q}_{2r}}}  &  {0} \cr 0  &  0  &  {{{\bar Q}_{3r}}} \cr } } \right],\quad$$

$$\hat A_r^l = \left[ {\matrix{ {{A^l}{T^l}S + {B^l}\Psi {Y_r}}  &  {{B^l}{\Phi ^1}{Y_r}}  &  {{B^l}{\Phi ^2}{Y_r}} \cr {{C^l}{T^l}S}  &  0  &  {0} \cr {\Psi {C^l}{T^l}S}  &  {{\Phi ^1}{S_{11}}}  &  {{\Phi ^2}{S_{11}}} \cr } } \right],\quad$$

$$\Xi = \left[ {\matrix{ {\sqrt {{Q_1}} {T^l}S}  &  0  &  {0} \cr 0  &  {\sqrt {{Q_2}} {S_{11}}}  &  {0} \cr 0  &  0  &  {\sqrt {{Q_3}} {S_{11}}} \cr } } \right],\quad$$

$${\bar Q_{1r}} = {({T^l}S)^T} + {T^l}S - {\tilde Q_{1r}},$$

$${\bar Q_{2r}} = {({S_{11}})^T} + {S_{11}} - {\tilde Q_{2r}},$$

$${\bar Q_{3r}} = {({S_{11}})^T} + {S_{11}} - {\tilde Q_{3r}},$$

$${Y_r} = {F_r}{S_{11}},$$

and we have Eq. (15) with $V({t_{k + n}})$ by Eq. (14). In addition, the related output-feedback gains in the control law (Eq. (9)) are given by

(17) $${F_r} = {Y_r}{S_{11}}^{ - 1}.$$

*Proof*: We can choose the token-dependent quadratic function that is defined by Eq. (14), *i.e*.,

(18) $$V(\chi ({t_{k + n}}|k)) = {\chi ^T}({t_{k + n}}|k){P_r}\chi ({t_{k + n}}|k),$$

where ${P_r} = diag\{ {P_{ir}}\} (i = 1,2,3)$ is the symmetric positive definite matrix that we wil design.

Calculate the difference of Eq. (14) along the trajectory of the system (Eq. (10)) yields.

$$\Delta V(\chi({t_{k + n}}|k)) = V(\chi({t_{k + n + 1}}|k)) - V(\chi({t_{k + n}}|k))$$

$$= {\chi ^T}({t_{k + n + 1}}|k){P_t}\chi ({t_{k + n + 1}}|k) - {\chi ^T}({t_{k + n}}|k){P_r}\chi ({t_{k + n}}|k)$$

(19) $$= {\chi ^T}({t_{k + n}}|k)(\bar A_r^T({t_k}){P_t}{\bar A_r}({t_k}) - {P_r})\chi ({t_{k + n}}|k)$$

We denote ${T^l} = [{({C^l})^T}{({C^l}{({C^l})^T})^{ - 1}}\quad {({C^l})^ \bot }]$, in which ${({C^l})^ \bot }$ represents the orthogonal basis of the null space for ${C^l}$, and we introduce a free matrix:

(20) $$S = \left[ {\matrix{ {{S_{11}}}  &  0 \cr  0  &  {{S_{22}}} \cr  } } \right],\quad$$

${S_{11}}$ is the arbitrary diagonal matrix, ${S_{22}}$ is the arbitrary matrix with appropriate dimension.

Substitute the following conditions $(i = 2,3)$

$${T^l}S + {({T^l}S)^T} - {\tilde Q_{1r}} - {({T^l}S)^T}\tilde Q_{1r}^{ - 1}{T^l}S\\ = - ({\tilde Q_{1r}} - {T^l}S)\tilde Q_{1r}^{ - 1}{({\tilde Q_{1r}} - {T^l}S)^T} \le 0,$$

$${S_{11}} + {({S_{11}})^T} - {\tilde Q_{ir}} - {({S_{11}})^T}\tilde Q_{ir}^{ - 1}{S_{11}}\\ = - ({\tilde Q_{ir}} - {S_{11}})\tilde Q_{ir}^{ - 1}{({\tilde Q_{ir}} - {S_{11}})^T} \le 0$$

into Eq. (16), we will get

(21) $$\left[ {\matrix{ {\check Q_r^l} & * & * & * \cr  {\hat A_r^l} & {{{\tilde Q}_t}} & * & * \cr  {\sqrt R \bar M} & 0 & {\rho I} & * \cr  \Xi & 0 & 0 & {\bar \rho I} \cr  } } \right]\quad \ge 0,$$

where

$$\check Q_r^l = \left[ {\matrix{ {{{({T^l}S)}^T}\tilde Q_{1r}^{ - 1}({T^l}S)} & 0 & 0 \cr  0 & {{{({S_{11}})}^T}\tilde Q_{2r}^{ - 1}({S_{11}})} & 0 \cr  0 & 0 & {{{({S_{11}})}^T}\tilde Q_{3r}^{ - 1}({S_{11}})} \cr  } } \right].\quad$$

If pre- and post-multiplying the following inequalities with diag $\{ {({T^l}S)^T},{({S_{11}})^T},{({S_{11}})^T},\underbrace {I,...,I}_7\}$ and its transpose, Eq. (21) will be obtained:

(22) $$\left[ {\matrix{ {\tilde Q_r^{ - 1}} & * & * & * \cr  {\bar A_r^l} & {{{\tilde Q}_t}} & * & * \cr  {\sqrt R M} & 0 & {\rho I} & * \cr  {\sqrt Q } & 0 & 0 & {\bar \rho I} \cr  } } \right]\quad \ge 0,$$

where $Q = diag\{ {Q_1},{Q_2},{Q_3}\}$,

$$\tilde Q_r^{ - 1} = \left[ {\matrix{ {\tilde Q_{1r}^{ - 1}} & 0 & {0} \cr 0 & {\tilde Q_{2r}^{ - 1}} & {0} \cr 0 & 0 & {\tilde Q_{3r}^{ - 1}} \cr } } \right],\quad$$

$$\bar A_r^l = \left[ {\matrix{ {{A^l} + {B^l}{F_r}\Psi {C^l}} & {{B^l}{F_r}{\Phi ^1}} & {{B^l}{F_r}{\Phi ^2}} \cr {{C^l}} & 0 & {0} \cr {\Psi {C^l}} & {{\Phi ^1}} & {{\Phi ^2}} \cr } } \right],\quad$$

$$M = {F_r}\left[ {\matrix{ {{\Psi _{\xi ({t_k})}}{C^l}} & {\Phi _{\zeta (\bar t_k^d)}^1} & {\Phi _{\zeta (\bar t_k^d)}^2} \cr } } \right].\quad$$

Because the system (Eq. (10)) is polytopic uncertain, *i.e*., inequality Eq. (22) is affine in $\Omega$, Eq. (22) means that

(23) $$\left[ {\matrix{ {\tilde Q_r^{ - 1}} & * & * & * \cr  {{{\bar A}_r}(k)} & {{{\tilde Q}_t}} & * & * \cr  {\sqrt R M} & 0 & {\rho I} & * \cr  {\sqrt Q } & 0 & 0 & {\bar \rho I} \cr  } } \right]\quad \ge 0.$$

According to the Schur Complement, we can deduce from Eq. (23) that

(24) $$\tilde Q_r^{ - 1} - \bar A_r^T({t_k})\tilde Q_t^{ - 1}{\bar A_r}({t_k}) - {M^T}{\rho ^{ - 1}}RM - {\rho ^{ - 1}}Q \ge 0.$$

Multiply both sides of Eq. (24) with $\rho \gt 0$ and define ${P_j} = \rho \tilde Q_j^{ - 1}(j \in \{ r,t\} )$, we can get

(25) $${P_r} - \bar A_r^T({t_k}){P_t}{\bar A_r}({t_k}) - {M^T}RM - Q \ge 0.$$

Pre- and post-multiplying Eq. (25) with ${\chi ^T}({t_k})$ and its transpose means

(26) $${\chi ^T}({t_k}){P_r}\chi ({t_k}) - {\nu _r}({t_k}) - {\omega _r} - {\chi ^T}({t_k})Q\chi ({t_k}) \ge 0.$$

where

$${\nu _r}({t_k}) = {({\bar A_r}({t_k})\chi ({t_k}))^T}{P_t}({\bar A_r}({t_k})\chi ({t_k})),$$

$${\omega _r} = {(M\chi ({t_k}))^T}R(M\chi ({t_k})).$$

It is noted that we have Eqs. (9), (10) and (19), then the condition (Eq. (15)) can be ensured by Eq. (26). So the proof is complete.

$Remark\;4.$ Despite the utilization of RR scheduling in the high-rate communication channel can avoid data conflict and network congestion, many side effects may happen because of the change of data transmission sequence. To deal with such a barrier, we employ the token-dependent Lyapunnov-like method. So the effect of the underlying RR scheduling can be reflected well in the design of the controller in this article. In Zou, Wang & Gao (2016a), Song et al. (2019), Zou et al. (2017b), to choose the token-dependent Lyapunov function for systems with various communication schedulings, a technical approach is proposed.

$Remark\;5.$ Of course, our proposed approach has some limitations. First, the RR protocol is a static protocol, only a part of the information can be updated at every time instant. So the amount of data in the system at each time will be relatively small, and the accuracy will also be affected. Second, the value of d cannot be too large in high-rate communication channels, which can be improved and optimized additionally.

Next, we will study the condition C2 of the terminal constraint set. Namely, all we have to do is to obtain the sufficient conditions, which can satisfy that the set $\Upsilon ({P_r},3\rho )$ is an RPI set.

Based on Definition 1, we need to satisfy the following two requirements to ensure the RPI set $\Upsilon ({P_r},3\rho )$:

$R1.$ at the time instant $n = 0$, the initial state belongs to the set $\Upsilon$, *i.e*.,

$$\chi{^T}({t_k})\tilde Q_r^{ - 1}\chi({t_k})\leq3;$$

$R2.$ the future states $\chi ({t_{k + n}}|k),n \gt 0$ are part of the set $\Upsilon$ too.

In the following content, we address above requirements one by one.

According to the Schur Complement, R1 holds if and only if

(27) $$\left[ {\matrix{ 3 & * \cr  {\chi ({t_k})} & {{{\tilde Q}_r}} \cr  } } \right]\quad \ge 0.$$

In addition, on account of Lemma 1, it is easy to see from Eq. (27) that

(28) $$V(\chi ({t_{k + n + 1}})) \le V(\chi ({t_{k + n}})) \le ... \le V(\chi ({t_k})) \le 3\rho$$

which means that all the future states $\chi ({t_{k + n}}|k)$ are part of the set $\Upsilon$ so long as $\Upsilon$ includes the initial state $\chi ({t_k})$. Thus we can also guarantee that the set $\Upsilon$ is an RPI set.

Up to now, by conditions Eqs. (16) and (27), the terminal constraint set can be ensured. That is to say, condition $\chi ({t_{k + n}}|k) \in \Upsilon ({P_r},3\rho )$ of OP1 is satisfied.

### Auxiliary optimization problems

For the system without constraints in this article, we discuss how to deal with the Op1.

Op1 is an optimization problem which includes parameter uncertainties over an infinite time horizon, it is very difficult to handle it directly. On the contrary, to find a sub-optimal solution, a certain auxiliary optimization issue will be proposed. We try to give such an auxiliary problem next.

We have Eq. (15) if the condition (Eq. (16)) holds. This means that $\chi (\infty |k) = 0$ and $V(\infty ) = 0$. Sum up both sides of Eq. (15) from $n = 0$ to $n = \infty$ and use Eq. (11) yields

(29) $${J_\infty }({t_k}) \le V({t_k}) = \chi{^T}({t_k}){P_r}\chi({t_k})\leq3\rho ,$$

which means

(30) $$\mathop {\max }\limits_{(A({t_k}),B({t_k}),C({t_k})) \in \Delta } {J_\infty }({t_k})\leq3\rho .$$

An upper bound of the objective function of Op1 is given.

On the basis of the above analysis, we are ready to present the following auxiliary optimization problem for the system without constraints:

$${\rm{Op2}}:\mathop {\min }\limits_{{{\tilde Q}_{ir}},{{\tilde Q}_{it}}(i = 1,2,3),(r,t) \in \,{\mathbb S},{F_r}} 3\rho ,$$

s.t. (Eqs. (16) and (27)).

The condition (Eq. (27)) is unable to be checked online because of the immeasurable state $x({t_k})$. We will handle the issue of unavailable states in constraint (Eq. (27)) next. Before continuing to work hard, the following significant assumption is showed.

$Assumption\;2.$ According to the initial state of the system (Eq. (1)), a known set is showed:

(31) $$x(0) \in \{ x({t_k})|{x^T}({t_k}){S^{ - 1}}x({t_k}) \le 1\} ,$$

matrix $S \gt 0$ can be predefied from practical experience.

$Lemma\;2.$ The system (Eq. (1)) is considered that it is controlled by Eq. (9), if there exist symmetric positive definite matrices ${\hat Q_{jr}}(j = 1,2,3)$, for the preseted Assumption 2, such that

(32) $$\left[ {\matrix{ 2  &  *  &  * \cr  {y({t_{k - 1}})}  &  {{{\tilde Q}_{2r}}}  &  * \cr  {\check y({t_{k - 1}})}  &  0  &  {{{\tilde Q}_{3r}}} \cr  } } \right]\quad \ge 0,$$

(33) $$\left[ {\matrix{ {{{\tilde Q}_r}}  &  * \cr  {\bar A_r^l{{\tilde Q}_r}}  &  {{{\hat Q}_r}} \cr  } } \right]\quad \ge 0,$$

(34) $${\tilde Q_{1r}} \ge {\hat Q_{1r}},{\tilde Q_{1r}} \ge S,{\tilde Q_{2r}} \ge {\hat Q_{2r}},{\tilde Q_{3r}} \ge {\hat Q_{3r}},$$

(35) $$\left[ {\matrix{ {{{\tilde Q}_r}}  &  *  &  * \cr  {[0,I,0]\bar A_r^l{{\tilde Q}_r}}  &  {2/3{{\hat Q}_{2r}}}  &  * \cr  {[0,0,I]\bar A_r^l{{\tilde Q}_r}}  &  0  &  {2/3{{\hat Q}_{3r}}} \cr  } } \right]\quad \ge 0.$$

hold, where ${\hat Q_r} \buildrel \Delta \over = \{ {\hat Q_{1r}},{\hat Q_{2r}},{\hat Q_{3r}}\}$ and ${\bar A^l}$ mean the vertices of $\bar A_{\rm\zeta (\it{Tk}^{\prime} + \it{n}){\rm }}(k)$, $l = 1,2,3,...,L,$ thus we can always guarantee condition (Eq. (27)).

*Proof*: By taking the similar to Ding et al. (2019), we can obtain the above lemma, which is thus omitted. So the proof is completed.

According to Schur Complement and Lemma 1, we can transform Eqs. (33) and (35) into the following conditions respectively $(l = 1,2,3,\ldots,L)$:

(36) $$\left[ {\matrix{ {\hat Q_r^l}  &  * \cr  {\hat A_r^l}  &  {{{\hat Q}_r}} \cr  } } \right]\quad \ge 0,$$

(37) $$\left[ {\matrix{ {\hat Q_r^l}  &  *  &  * \cr  {{{\scr{X}}_r}}  &  {2/3{{\hat Q}_{2r}}}  &  * \cr  {{\scr{Y}_r}}  &  0  &  {2/3{{\hat Q}_{3r}}} \cr  } } \right]\quad \ge 0,$$

where

$${{\scr{X}}_r} = \left[ {\matrix{ {{C^l}{T^l}S}  &  0  &  {0} \cr } } \right],$$

$${{\scr{Y}}_r} = \left[ {\matrix{ {\Psi {C^l}{T^l}S}  &  {{\Phi ^1}{S_{11}}}  &  {{\Phi ^2}{S_{11}}} \cr } } \right].$$

For the solvability, we can transform the issue Op2 into the following approximate optimization based on Lemma 2 and Assumption 2:

$${\rm{Op3}}:\mathop {\min }\limits_{{{\tilde Q}_{ir}} \gt 0,{{\tilde Q}_{it}} \gt 0,{{\hat Q}_{ir}} \gt 0(i = 1,2,3),(r,t) \in {\mathbb S} \times {\mathbb S}, {F_r}} 3\rho ,$$

s.t. Eqs. (16), (31), (32) and Eqs. (34), (36), (37).

### Feasibility and stability

We will make the feasibility of the proposed issues clear. And to move forward a single step, we will show the stability of the system (Eq. (1)), which is controlled by Eq. (9).

$Theorem\;1.$ Let us give the symmetric positive definite matrices ${Q_1},{Q_2},{Q_3}$ and *R*. We take the system (Eq. (1)) controlled by Eq. (9) into account. If a feasible solution for the optimization issue Op3 at the initial time instant ${t_k}$ exists, then at any future time instant $t \gt {t_k}$, the corresponding feasible solution also exists. Beside, the closed-loop system is asymptotically stable and Eq. (9) determines feedback gains.

*Proof*: (1) $Feasibility$. At the initial time instant ${t_k}$, let us assume that the optimization issue Op3 is feasible. For the time instant ${t_{k + n}},n \ge 1$ in the future, our need is proving that the issue Op3 is feasible too. It is not hard to see that only the condition (Eq. (16)) depends on the states, but other conditions are feasible at the time instant $t \gt {t_k}$ in the future so long as they are feasible at the time instant ${t_k}$. To this extent, we only need to prove that the condition (Eq. (16)) is feasible at the future time instant. Namely, the feasibility of the condition (Eq. (27)) in Op2 need to be proved. From Eqs. (1) and (10), we can get the following relations:

(38) $$\chi ({t_{k + n}}|k + n) = \chi ({t_{k + n}}),n \ge 1$$

(39) $$\chi ({t_{k + 1}}|k) = {\bar A_{\xi ({t_k})}}({t_k})\chi ({t_k}|{t_k}),$$

(40) $$\chi({t_{k + 1}}) = {\bar A_{\xi ({t_k})}}({t_k})\chi({t_k})\quad for\;some\;{\bar A_{\xi ({t_k})}}({t_k}) \in \Delta$$

Based on the property of the RPI set, we have

(41) $${\chi ^T}({t_{k + 1}}|k)Q_t^{ - 1}\chi ({t_{k + 1}}|k)\lt{\chi ^T}({t_k})Q_r^{ - 1}\chi ({t_k})\lt3.$$

Using Eqs. (39) and (40), we can get the condition from Eq. (41):

(42) $${\chi ^T}({t_{k + 1}})Q_t^{ - 1}\chi ({t_{k + 1}})\lt3.$$

This indicates that the condition (Eq. (27)) is feasible at the time instant ${t_{k + 1}}$. In addition, this process can continue at any time ${t_{k + 2}},{t_{k + 3}},...$ in the future.

(2) $Stability$. We need to construct a decreasing quadratic function $\bar V(\chi ({t_k})) = {\chi ^T}({t_k}){P^*}_{\xi ({t_k})}\chi ({t_k})$ to prove that the system (Eq. (1)) is asymptotically stable, the subscript “*” means the optimal solution of issue Op3 at the time instant ${t_k}$. Based on the above Feasibility, one has

(43) $${\chi ^T}({t_{k + 1}}){P^*}_{\xi ({t_{k + 1}})}\chi ({t_{k + 1}}) \le {\chi ^T}({t_{k + 1}}){P^*}_{\xi ({t_k})}\chi ({t_{k + 1}})\lt{\chi ^T}({t_k}){P^*}_{\xi ({t_k})}\chi ({t_k}),$$

where ${P_i}(i \in \{ \xi ({t_k}),\xi ({t_{k + 1}})\} )$ without the subscript “*” means the feasible solution.

The quadratic function $\bar V(\chi ({t_k}))$ is strictly decreasing, we can end the proof of the theorem.

### MPC under RR scheduling with hard constraints

In this part, the MPC issue for the polytopic systems with hard constraints under RR scheduling is to be handled, which based on the establishments made before. After that, we obtain several sufficient conditions. At the end, subject to certain conditions, an algorithm is proposed for addressing an online optimization issue.

### Controller design under RR scheduling

First, to ensure the hard constraints for the inputs and states (Eq. (3)), several inequalities are proposed. Afterward, in the framework of MPC for the system with constraints, the optimization issue is presented to design the controllers, and the related algorithm is proposed.

$Lemma\;3.$ If symmetric positive definite matrices ${S_{11}},{\tilde Q_{1r}}$ and matrices ${Y_r}$ exist, hard constraints for the inputs and the states (Eq. (3)) are met. Then, for any $(r,t) \in \mathbb R \times \mathbb R$, the conditions

(44) $$\left[ {\matrix{ I  &  * \cr  {Y_r^T}  &  {{{\bar u}^2}{S_{11}}} \cr  } } \right]\quad \ge 0,$$

(45) $$\left[ {\matrix{ I  &  * \cr  {{{\tilde Q}_{1r}}}  &  {{{\bar x}^2}{{\tilde Q}_{1r}}} \cr  } } \right]\quad \ge 0.$$

hold.

*Proof*: In view of the constraint on the input predictions, for any $n \ge 0$, we get from Eq. (9) that

(46) $$\matrix{ {|{{[u({t_{k + n}}|k)]}_c}{|^2}} \hfill  &  { = |{r_c}({F_r}{\check y}({t_{k + n}}|k)){|^2}} \hfill \cr {} \hfill  &  { = |{r_c}{Y_r}{S_{11}}^{ - 1}{\check y}({t_{k + n}}|k){|^2}} \hfill \cr {} \hfill  &  { = |{r_c}{Y_r}{S_{11}}^{ - 1/2}{S_{11}}^{ - 1/2}{\check y}({t_{k + n}}|k){|^2}} \hfill \cr {} \hfill  &  { \le \parallel {r_c}{Y_r}{S_{11}}^{ - 1/2}{\parallel ^2}\parallel {S_{11}}^{ - 1/2}{\check y}({t_{k + n}}|k){\parallel ^2}} \hfill \cr {} \hfill  &  {(from\;Cauchy\;Schwarz\;inequality)} \hfill \cr {} \hfill  &  { \le \parallel {r_c}{Y_r}{S_{11}}^{ - 1/2}{\parallel ^2}} \hfill \cr {} \hfill  &  { = {r_c}({Y_r}{S_{11}}^{ - 1}Y_r^T)r_c^T} \hfill \cr {} \hfill  &  { \le {{\bar u}^2}} \hfill \cr }$$

${r_c}$ is the $c$th row of an ${n_u}$-ordered identity matrix. Eq. (46) holds if and only if Eq. (44) holds according to the Schur Complement.

Afterward, in terms of the constraint for the state predictions, we can get the following by the similar technique presented above:

(47) $$\matrix{ {|{{[x({t_{k + n}}|k)]}_d}{|^2}} \hfill  &  { = |{r_d}{{\tilde Q}_{1r}}^{1/2}{{\tilde Q}_{1r}}^{ - 1/2}[x({t_{k + n}}|k)]{|^2}} \hfill \cr {} \hfill  &  { \le \parallel {r_d}{{\tilde Q}_{1r}}^{1/2}{\parallel ^2}\parallel {{\tilde Q}_{1r}}^{ - 1/2}[x({t_{k + n}}|k)]{\parallel ^2}} \hfill \cr {} \hfill  &  {(from\;Cauchy\;Schwarz\;inequality)} \hfill \cr {} \hfill  &  { \le \parallel {r_d}{{\tilde Q}_{1r}}^{1/2}{\parallel ^2}} \hfill \cr {} \hfill  &  { = {r_d}{{\tilde Q}_{1r}}r_d^T} \hfill \cr {} \hfill  &  { \le {{\bar x}^2}} \hfill \cr }$$

${r_d}$ is the $d$th row of an ${n_x}$-ordered identity matrix. Eq. (47) holds if and only if Eq. (45) holds according to the Schur Complement.

For the constrained system under RR scheduling in the high-rate communication channel, according to Lemma 3, we can obtain a further auxiliary optimization issue by

$${\rm{Op4}}:\mathop {\min }\limits_{{{\tilde Q}_{ir}} \gt 0,{{\tilde Q}_{it}} \gt 0,{{\hat Q}_{ir}} \gt 0(i = 1,2,3),(r,t) \in \,{\mathbb S} \times {\mathbb S},{F_r}} 3\rho ,$$

s.t. (Eqs. (16), (31), (32)) and (Eqs. (34), (36), (37), (44), (45)).

According to the above discussions, we get ready to show the following theorem such that the underlying system (1) with hard constraints under RR scheduling is asymptotically stable.

$Theorem\;2.$ Under the RR scheduling in the high-rate communication channel, Eqs. (1) and (3) are controlled by Eq. (9), the system (Eq. (1)) with hard constraints is considered. At the initial time instant ${t_k}$, if the optimization problem Op4 is feasible, then for the time instants $t \gt {t_k}$ in the future, the optimization issue Op4 is feasible too. In addition, the closed-loop system is stable because of the feedback gains ${F_r} = {Y_r}({t_k}){S_{11}}^{ - 1}.$

*Proof*: The proof procedure is omitted because it is similar to the process of proof in Theorem 1.

### Algorithm of MPC for constrained system under RR scheduling

In this part, considering the RMPC strategy, the algorithm for the systems with constraints under RR scheduling in the high-rate communication channel is to be shown.

**Algorithm**:
$Off - linepart:$
Choose an initial state $\chi(0) = [x(0)\;y( - 1)\;\check y( - 1)]$ and proper matrix S, $x(0) \in \{ x({t_k})|{x^T}({t_k}){S^{ - 1}}x({t_k}) \le 1\}$ is feasible at $k = 0$.
$On - linepart:$
$Step\;1$. Firstly, at the time instant ${t_k}$, address the optimization issue Op4 to get the controller gain ${F_r}$ by the RR scheduling and the parameters in Off-line part.
$Step\;2$. Secondly, calculate ${F_r} = {Y_r}({t_k}){S_{11}}^{ - 1}$ and act $u({t_k}) = {F_r}\check y({t_k})$ on the plant and return to $Step\;1$.

$Remark\;6.$ For discrete time linear systems with polytopic uncertainties, the RMPC issue is handled under the RR scheduling in the high-rate communication channel. The main unique features of our results are as follows: (1) because the communication capacity of the network channel is limited, the RR scheduling is adopted to handle the RMPC issue; (2) due to the coupling between the high-rate communication channels and the RR scheduling, the complicated transmission mechanism of the sensors is modeled by a periodic sequence; (3) the optimization issue Op4 is produced to get a certain upper bound for quadratic cost function; (4) an online RMPC algorithm is put forward to get several controllers, which make the system mentioned before asymptotically stable.

## Illustrative Example

In this part, two examples are proposed to test and verify the validity of the MPC strategy that we propose in this article. The system in a distillation process has reflux and boil-up radio, two manipulated variables; top and bottom composition, and two controlled variables.

Example 1: A continuous-time distillation process system is borrowed from Al-Gherwi, Budman & Elkamel (2011), which consists of two manipulated variables, reflux and boil-up ratio and two controlled variables, top and bottom composition. Since the RMPC problem addressed in this article is for networked control systems where data are transmitted *via* the high-rate network and the control inputs, the analog signal should be converted into the digital signal, that is to say, the continuous-time system needs to be discretized. By selecting the same sampling period, we obtain the discrete-time system from Al-Gherwi, Budman & Elkamel (2011). From the practical viewpoint, we consider the parameter uncertainties in system matrices. Choose the system model:

$$x({t_{k + 1}}) = \left[ {\matrix{ {0.9481} & {0} \cr 0 & {0.9481} \cr } } \right]x({t_k})\\ + ( - 14)*\left[ {\matrix{ {0.512} & {0.015} \cr {0.086} & {0.469} \cr } } \right]u({t_k}) \buildrel \Delta \over = Ax({t_k}) + Bu({t_k}),$$

$$y({t_k}) = \left[ {\matrix{ {1.5}  &  {0} \cr 0  &  {1.5} \cr } } \right]x({t_k}) \buildrel \Delta \over = Cx({t_k})$$

with the initial value

$$x(0) = \left[ {\matrix{ 1 \cr  { - 1} \cr  } } \right],\quad y( - 1) = \left[ {\matrix{ { - 3} \cr  3 \cr  } } \right]and\quad \check y( - 1) = \left[ {\matrix{ {0.5} \cr  {0.5} \cr  } } \right].\quad$$

After that, to better satisfy the polytopic uncertainties requirements of the actual system, the system parameters are presented:

$${A^{(1)}} = {A^{(2)}} = A = \left[ {\matrix{ {0.9481} & {0} \cr 0 & {0.9481} \cr } } \right],$$

$${C^{(1)}} = {C^{(2)}} = \left[ {\matrix{ {1.5} & {0} \cr 0 & {1.5} \cr } } \right],$$

$${Q_1} = {Q_2} = {Q_3} = \left[ {\matrix{ 3 & {0} \cr 0 & {3} \cr } } \right],\quad R = 0.01*\left[ {\matrix{ {0.05} & {0} \cr 0 & {0.05} \cr } } \right].$$

We give the upper bounds of input and state by $\bar u = 50$ and $\bar x = 900$, respectively. The weighting matrix is defined as

$$S = \left[ {\matrix{ 0 & {0.9} \cr {0.9} & {0} \cr } } \right].$$

**Figure 2:**
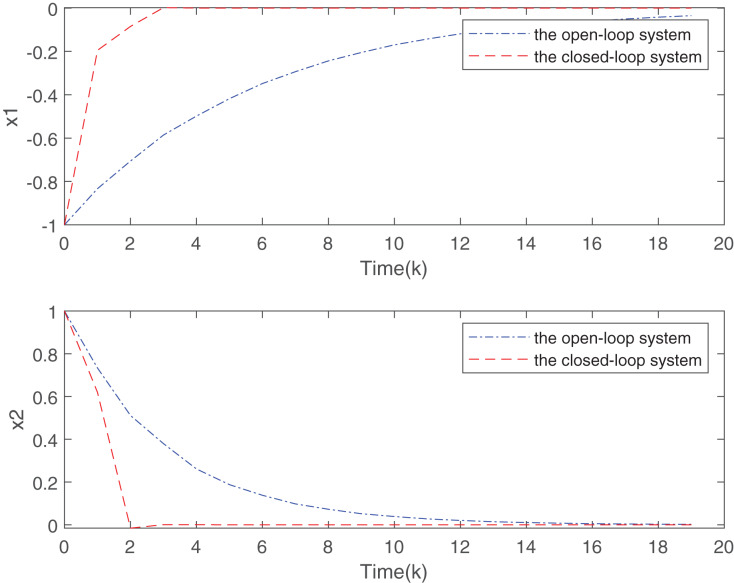
State responses for the open-loop and closed-loop systems.

**Figure 3:**
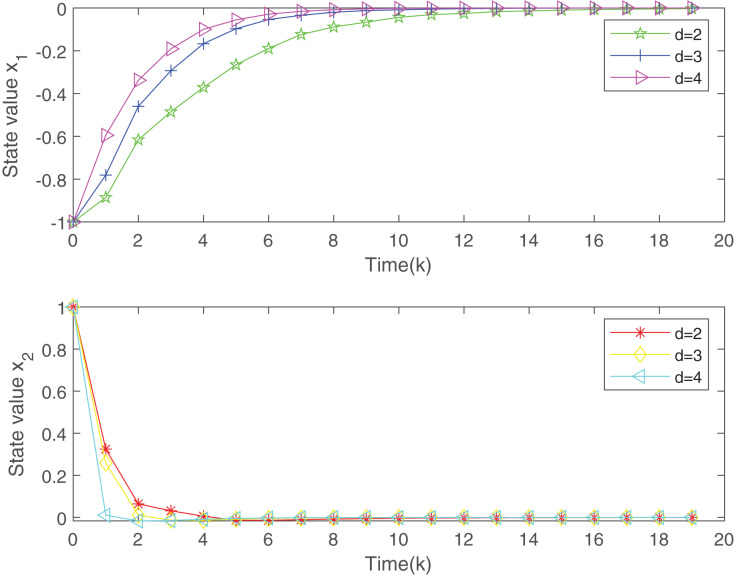
The evolution of states of the closed-loop system.

Two sensor nodes transmit the measurements to the controller through a sharing network with high-rate communication channels. At the initial time instant, the sensor node 1 is executed and move ahead in its order based on the RR scheduling. The simulation results are presented in Figs. 2 and 3.

**Figure 4:**
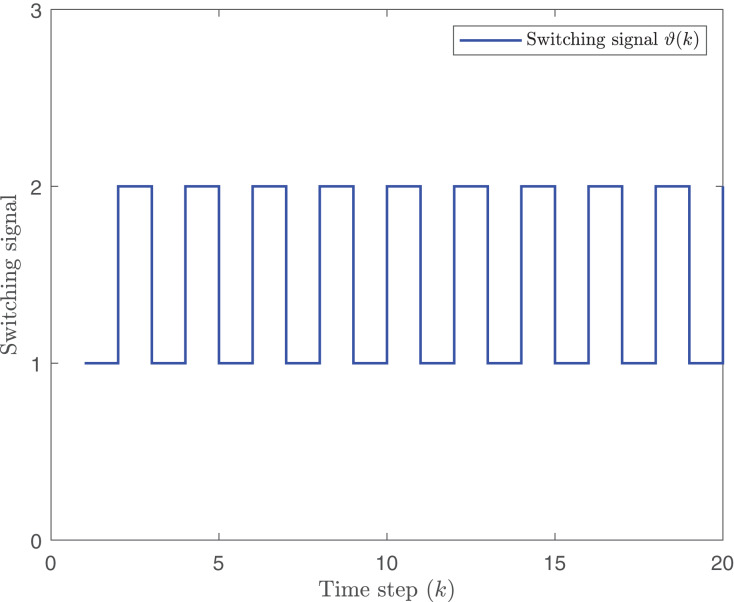
Switching signal.

Figure 2 plots the trend of states for system without the designed controller and system with the controller. From Fig. 2, it is easy to find that the closed-loop system addressed is more stable than the open-loop system under the proposed static MPC algorithm. From the Fig. 3, with the increase of $d$, the system is more stable. Figure 4 gives the evolution of $\vartheta (k)$.

Example 2: The second example considers an unstable system without control. The parameters are given as follows:

$${A^{(1)}} = A = \left[ {\matrix{ 1 & {0.1} \cr { - 0.1} & { - 0.1} \cr } } \right],\quad {A^{(2)}} = \left[ {\matrix{ {1.1} & {0.15} \cr { - 0.1} & { - 0.2} \cr } } \right],$$

$${B^{(1)}} = {B^{(2)}} = \left[ {\matrix{ {1.9247} & {0.5124} \cr {0.1862} & {1.1692} \cr } } \right],$$

$${C^{(1)}} = {C^{(2)}} = \left[ {\matrix{ {1.5} & {0} \cr 0 & {1.5} \cr } } \right].$$

**Figure 5:**
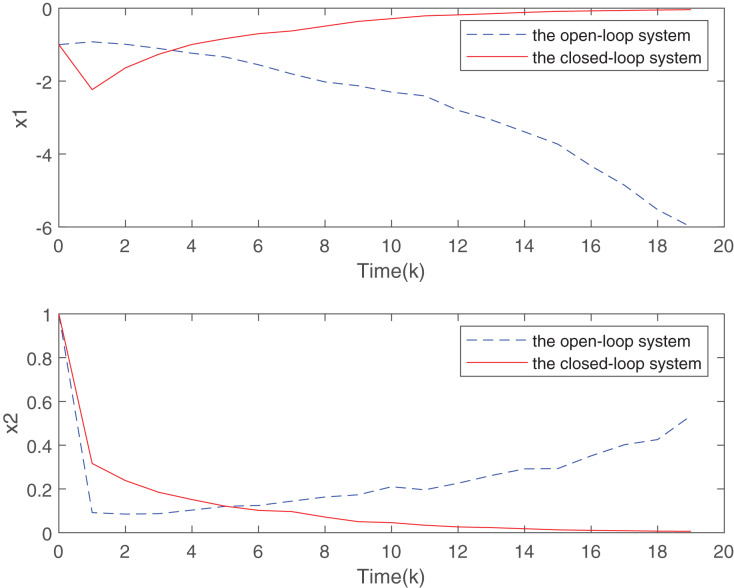
State responses for the open-loop and closed-loop systems.

**Figure 6:**
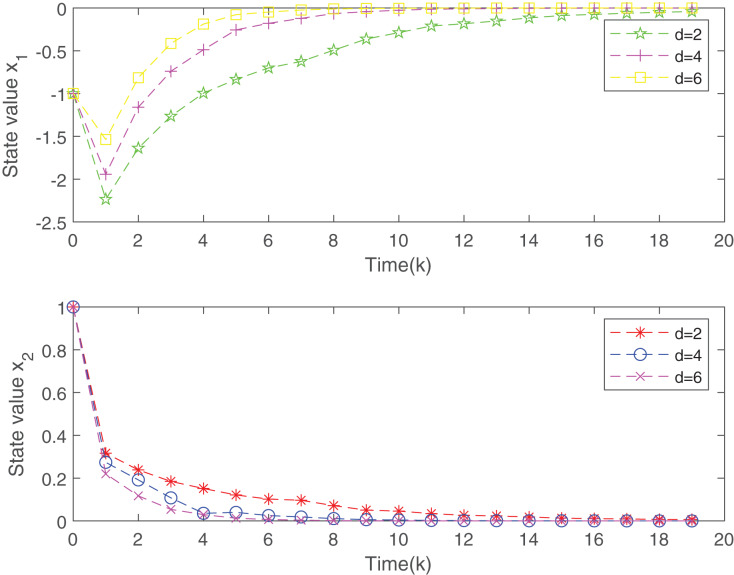
The evolution of states of the closed-loop system.

**Figure 7:**
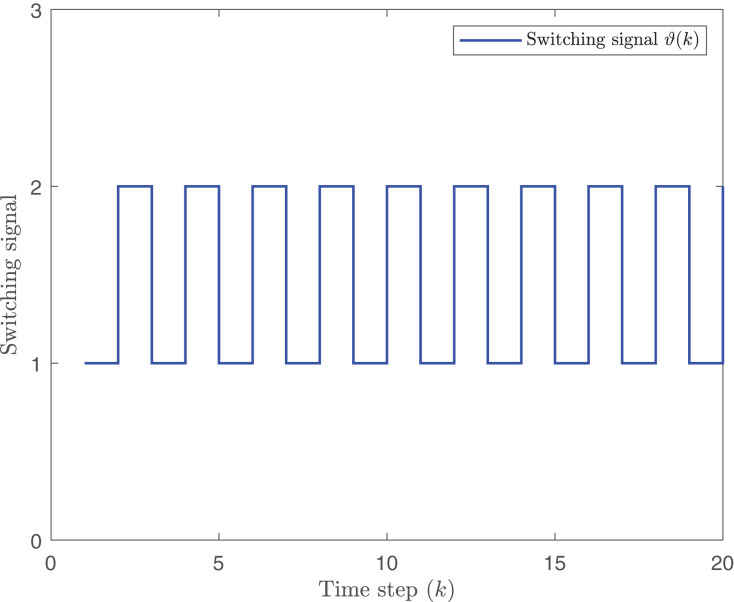
Switching signal.

Figure 5 plots the trend of the states for the system without the designed controller and system with the controller. From Fig. 5, we can see that the system is stable under the designed RMPC controller, which shows that the presented RMPC scheme is necessarily effective. As can be seen from the Fig. 6, system with the controller tends to stabilize more quickly owing to $d$ gradually increasing. Figure 7 plots the evolution of $\vartheta (k)$.

## Conclusions

In this article, the RMPC problem has been investigated for the discrete-time polytopic uncertain system unmeasurable states under RR scheduling in the high-rate communication channel. For the sake of handling with the limited communication bandwidth issue, we introduce the round-robin scheduling to evenly distribute the communication resource with limits, *e.g*., only one node has the privilege of establishing a connection to the sharing high-rate communication network at the current instant, the other nodes will retain information of the last time according to the zero-order holders (ZOHs). Due to the high-rate communication scheduling, data information can be more accurate. Drawing support from the Lyapunov stability theory, the RMPC controllers have been designed by handling the optimization issue, and we have obtained sufficient conditions. After that, an upper-bound of the quadratic cost function has been achieved. In the end, two simulation examples have been used for demonstrating the effectiveness of the proposed RMPC algorithm. In our future work, we might extend the established results on RMPC to some other communication protocols such as the stochastic communication protocol, the event-triggering scheme and so on.

## Supplemental Information

10.7717/peerj-cs.1269/supp-1 Supplemental Information 1 Two simulation examples have been used for demonstrating the effectiveness of the proposed MPC algorithm.

## Additional Information and Declarations

### Data Availability

The following information was supplied regarding data availability: The code is available in the Supplemental File.
